# Characterization of Thermal and Time Exposure to Improve Artificial Diet for Western Corn Rootworm Larvae

**DOI:** 10.3390/insects12090783

**Published:** 2021-09-01

**Authors:** Man P. Huynh, Adriano E. Pereira, Ryan W. Geisert, Michael G. Vella, Thomas A. Coudron, Kent S. Shelby, Bruce E. Hibbard

**Affiliations:** 1 Division of Plant Science & Technology, University of Missouri, Columbia, MO 65211, USA; pereiraa@missouri.edu (A.E.P.); geisertblue@gmail.com (R.W.G.); 2 Department of Plant Protection, Can Tho University, Can Tho 900000, Vietnam; 3 Frontier Scientific Services, Newark, DE 19711, USA; mvella@fsiag.com; 4 Biological Control of Insects Research Laboratory, USDA-Agricultural Research Service, Columbia, MO 65203, USA; coudront@missouri.edu (T.A.C.); kent.shelby@usda.gov (K.S.S.); 5 Plant Genetics Research Unit, USDA-Agricultural Research Service, Columbia, MO 65211, USA; bruce.hibbard@usda.gov

**Keywords:** *Diabrotica virgifera*, corn rootworm, WCRMO-2, diet processing, heating

## Abstract

**Simple Summary:**

The western corn rootworm is a highly adaptive pest that has evaded nearly all management tactics developed to date. Antibiotics have been utilized in rootworm diets to mitigate bacterial contamination. However, antibiotic ingestion necessarily alters rootworm gut microbiota, clouding the outcome of diet toxicity bioassays used in determination of rootworm susceptibility to insecticides. Rapid heating, or pasteurization, is one of the most widely applied techniques to alleviate microbial contamination and could eliminate antibiotics from the diet. We characterized effects of temperatures and time intervals of thermal exposure on quality of rootworm diet by measuring larval weight, molting, and survival. Our results demonstrated non-linear effects of thermal exposure on the performance of diet, whereas no impacts were observed on the exposure intervals evaluated. These findings will guide the continued development of sterilized rootworm diets, facilitating mass production and provide insights into the design of diets for other insects.

**Abstract:**

The western corn rootworm (WCR), *Diabrotica virgifera* LeConte, is the most serious pest of maize in the United States. In pursuit of developing a diet free of antibiotics for WCR, we characterized effects of thermal exposure (50–141 °C) and length of exposure on quality of WCRMO-2 diet measured by life history parameters of larvae (weight, molting, and survival) reared on WCRMO-2 diet. Our results indicated that temperatures had non-linear effects on performance of WCRMO-2 diet, and no impacts were observed on the length of time exposure. The optimum temperature of diet processing was 60 °C for a duration less than 30 min. A significant decline in development was observed in larvae reared on WCRMO-2 diet pretreated above 75 °C. Exposing WCRMO-2 diet to high temperatures (110–141 °C) even if constrained for brief duration (0.9–2.3 s) caused 2-fold reduction in larval weight and significant delays in larval molting but no difference in survival for 10 days compared with the control diet prepared at 65 °C for 10 min. These findings provide insights into the effects of thermal exposure in insect diet processing.

## 1. Introduction

The western corn rootworm (WCR), *Diabrotica virgifera virgifera* LeConte (Coleoptera: Chrysomelidae), is the most serious pest of maize in the United States and some parts of Europe [1], causing 1 to 2 billion dollars (USD) in losses and control costs to U.S. maize growers each year [2]. Most damage associated with this species is the result of larval feeding on maize roots [3,4], though yield reduction of maize can result from adult feeding on silks, pollen, kernels, and foliage of maize plants [5]. Management of WCR has been a challenge because this highly adaptive insect has evolved resistance to several management strategies, including chemical insecticides [6,7,8,9], transgenic maize hybrids expressing insecticidal crystalline toxins from *Bacillus thuringiensis* (Bt) Berliner [10,11,12,13,14,15], and cultural control techniques, such as crop rotation [16,17].

Given a history of developing resistance to nearly every management tactic utilized for managing WCR, a logical concern exists that WCR will possibly develop resistance to newer management tactics. To slow resistance development of this pest, the U.S. Environmental Protection Agency (EPA) has mandated monitoring resistance programs that involve annual collections of insect populations in regions of high adoption of the targeted trait followed by bioassays to determine potential reduction in susceptibility attributable to resistance development [18]. Diet assays, whereby insects are exposed to toxins in an artificial diet, that can be used in conjunction with on-plant assays to evaluate the susceptibility of WCR to insecticides are critical components of the resistance-monitoring programs [19,20,21].

An artificial diet capable of supporting WCR larval growth and development similar to those fed on maize roots would be greatly beneficial for research programs. Artificial diet development for *Diabrotica* spp. was initially conducted on the southern corn rootworm, *Diabrotica undecimpunctata howardi* Barber, as a model species [22,23] because diet work began prior to availability of the non-diapausing strain of WCR. An attempt to develop a diet for WCR rearing occurred in 2002 [24]. Later, Huynh et al. [25] developed an improved WCR diet (WCRMO-1) that was an optimization of the ingredients in the initial WCR diet [24]. The WCRMO-1 formulation is compatible with each of the four marketed Bt toxins targeting WCR [26]. However, both published WCR diets [24,25] require maize root powder, which is not available for purchase, thereby limiting the practical use of the diets. Recently, Huynh et al. [27] successfully developed a WCR diet without maize root powder (WCRMO-2) that supports performance of WCR larvae equal to or better than that of publicly available formulations [27,28]. The WCRMO-2 formulation was specifically designed to require only commercially available ingredients. This formulation supported approximately 97% of larvae for survival, molting, and increased larval weight gain after 10 days of feeding by 4-fold compared with the WCRMO-1 diet [27]. Both WCRMO-1 and WCRMO-2 diets have essentially zero microbial contamination [27], similar to the four proprietary diets previously used for WCR bioassays [26,28], through clean laboratory practices described previously [25]. The WCRMO-2 diet is now available commercially (WCRMO-2, Frontier Scientific Services, Newark, DE, USA).

Antibiotics are commonly used in insect diets for preventing bacterial contamination. Ingestion of antibiotics in insect diets has been reported to alter response of lepidopteran insects to Bt proteins due to its negative effects on insect gut microbiomes [29,30]. In fact, Paramasiva, Sharma, and Krishnayya [29] manipulated antibiotics to suppress gut microflora in the cotton bollworm (*Helicoverpa armigera* Hübner) and found that *H. armigera* larvae fed on larval diet with antibiotics had lower susceptibility to a commercial formulation of Bt and purified δ-endotoxins Cry1Ab and Cry1Ac compared with *H. armigera* larvae fed on larval diet without antibiotics. Additionally, pretreatment of *H. armigera* larvae with antibiotics to eliminate the gut microbes resulted in a decrease in larval mortality and an increase in the larval weight gain when *H. armigera* larvae were exposed to activated Cry1Ac, Bt formulation, and transgenic cotton [30]. In WCR, antibiotics have been reported to cause effects on the indigenous symbionts that contribute the development of resistance in WCR to crop rotation [31]. Differences in the abundance of multiple bacterial taxa (e.g., *Acinetobacter* sp., *Pseudomonas* sp., *Enterobacter* sp., *Lactococcus* sp.) in WCR gut microbiota were correlated with increased resistance to soybean-defense compounds. Gut microbiota of WCR derived from rotation-resistant populations and wild-type populations had differences in the abundance of *Klebsiella* sp., *Stenotrophomonas* sp., *Enterobacter* sp., and *Lactococcus* sp. [31]. The authors suppressed gut microbiota of rotation-resistant WCR by antibiotic treatments, finding that the resistance to the soybean-defensive compound of the resulting rotation-resistant WCR was reduced to a level similar to that of wild-type WCR. Paddock et al. [32] reported that feeding on maize expressing a Bt toxin resulted in a shift in the gut microbiota in susceptible WCR larvae but led to no changes in the bacterial community within resistant insects. Currently, published diet formulations for *Diabrotica* spp. contain antibiotics (streptomycin and chlortetracycline) [22,24,27,33] that are widely used in other insect diets as the important antibacterial agents [34]. It is likely that these antibiotics have potential impacts on WCR gut microbiomes, though these effects of antibiotics on WCR larvae are not adequately characterized. The use of a diet free from antibiotics for WCR bioassays would likely provide more accurate phenotypic picture of test populations, as it relates to susceptibility to Bt toxins and other insecticide compounds. Therefore, development of a WCR diet free of antibiotics would facilitate resistance-monitoring programs as well as other research programs of this pest.

One of the most widely used techniques to alleviate microbial contamination in insect diets is thermal treatment as a means of diet preservation [34]. During diet processing, agar-based diets are often exposed to elevated temperatures ranging from mild blanching (50–100 °C) to high temperatures above the boiling point of water. These high-thermal treatments are typically manipulated with pressure, which can lead to the destruction of microbial contaminants. Flash sterilization is one of the heating techniques that utilizes high temperatures for a brief time of thermal exposure (less than 1 min) that sufficiently kills microbial contaminants in the diet [34] while minimizing the effects of overheating on heat-sensitive nutrients (e.g., vitamins, amino acids, or lipids) [35]. As a step toward development of a diet free of antibiotics for WCR, we investigated the effects of high temperatures (80–141 °C) for short time exposure (0.8–2.3 s) on quality of WCRMO-2 diet using a flash sterilization approach. Additionally, since the effects of using relatively low temperatures on the performance of insect diets have not been adequately explored, we further characterized the effects the mild thermal exposure (50–80 °C) and extended time of thermal exposure (5–30 min), which are commonly used in insect diet preparation, on the quality of WCRMO-2 diet. The quality of WCRMO-2 diet was assessed via the evaluation of life history parameters (weight, molt, and survival) of WCR larvae reared on the diets for 10 days.

## 2. Materials and Methods

### 2.1. Insects and Egg Treatment

Eggs of non-diapausing WCR populations were obtained from the USDA-ARS- Plant Genetics Research Unit (PGRU) laboratory in Columbia, MO. Egg plates containing WCR eggs and soil in Petri dishes were incubated at 25 °C in complete darkness until ~5% of eggs were hatched. Subsequently, the eggs were washed from soil with tap water and then were surface-sterilized using undiluted Lysol (Clean & Fresh Multi-Surface Cleaner, Reckitt Benckiser, Parsippany, NJ, USA) for 3 min and followed by 10% formaldehyde (HT501128, Sigma Aldrich, St. Louis, MO, USA) for 3 min, as described previously [24,36]. The eggs were dispensed onto coffee filter paper (Pure Brew, Rockline Industries, Sheboygan, WI, USA) placed inside a 16 oz. cup (11.7 × 7.62 × 9.6 cm, DM16R-0090, Solo Cup Company, Lake Forest, IL, USA) with a lid (LG8RB-0090, Solo Cup Company) using a 1-mL disposable pipette (13-711-9a, Fisher Scientific, Pittsburg, PA, USA). The eggs were then incubated at 25 °C in darkness. Larvae that hatched within 24 h were used for insect bioassay.

### 2.2. Experimental Approach

A series of experiments were performed to determine the effects of thermal and time exposure on the quality of WCRMO-2 diet. WCRMO-2 diet was made using WCRMO-2 dry mix (WCRMO-2, Frontier Scientific Services) at different temperatures and varying lengths of time of exposure to the different temperatures. The WCRMO-2 dry mix (Frontier Scientific Services) consists of ingredients, diet preservatives (antifungal and antibacterial agents), and agar that were published previously [27]. The quality of WCRMO-2 diet was evaluated by life history parameters of WCR larvae (weight, molt, and survival) reared on the diet treatments for 10 days.

Two-level factorial designs were constructed to determine the effects of two factors (temperature and time of thermal exposure) on the quality of WCRMO-2 diet. Two experiments were performed to evaluate high temperatures and brief time of thermal exposure (80–141 °C and 0.8–2.3 s) and mild temperatures and extended time of thermal exposure (50–80 °C and 5–30 min). The high temperature and the brief time of thermal exposure (141 °C for 2 s) was previously used for flash sterilization of a larval diet for *Trichoplusia ni* (Hubner) (Lepidoptera: Noctuidae) [37]. Since preliminary observations indicated that time of thermal exposure up to 10 min at 65 °C had no effects on the quality of WCRMO-2 diet, the extended time of thermal exposure up to 30 min was selected to further determine the effects of the longer duration of thermal exposure. The experimental designs for each experiment were generated with Design-Expert (Stat-Ease, Inc., Minneapolis, MN, USA). All designs consisted of 11 design points (diet treatments at different thermal exposure and time of exposure) with 5 model, 3 lack of fit, and 2 pure error degrees of freedom. In all experiments, WCRMO-2 diet made using the standard protocol according to the manufacturer’s procedure, with a temperature of 65 °C and time of exposure for 10 min [27], was included as a control (Table 1).

A flash sterilization system (Frontier Scientific Services) was utilized to produce WCRMO-2 diet at high thermal exposure (80–141 °C) and short time exposure (0.8–2.3 s). We observed consistent failures in the ability of the powdered agar to melt into solution during the brief time and rather high thermal exposure experienced in treatments with temperatures less than 88 °C (Table 1). This is likely due to the physical limitations of the agar (7060, Frontier Scientific Services) used to operate adequately at the mild temperatures tested. This resulted in a “soft-set” of the media, providing an inadequate matrix to support larval growth, leading to the death of the majority of larvae and increased bacterial contamination after 4 days post infestation. Low-melt agar (Frontier Scientific Services) was tested as a substitution of the agar used initially. However, the “soft-set” of the media was observed when the media were prepared with the low melt agar at the temperatures ≤ 65 °C for small windows of time (0.9–2.1 s). To further study the effects of mild thermal exposure (50–80 °C) on the quality of WCRMO-2 diet, an alternative approach involved completely melting the agar using a microwave (51101BZ, Hamilton Beach, Glen Allen, VA, USA) and cooling it to designed mild temperatures prior to adding it to the treated media was evaluated. The temperatures were then held for designed time exposure (5–30 min) using a hot plate (Cimarec^TM^, Thermo Scientific, Waltham, MA, USA) (Table 1).

## 3. Diet Preparation

**Figure 1:**
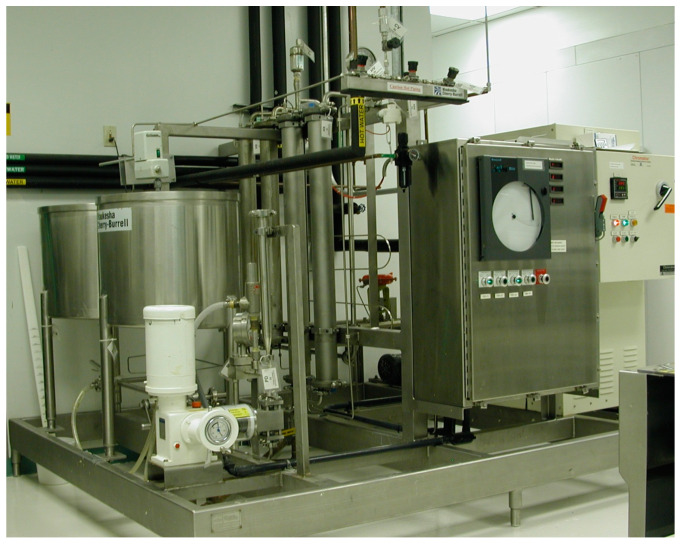
Frontier Scientific’s flash sterilization system used for the processing of insect media. From left to right: diet tank, pump, processing tubes, coiled spiraltherms, and control station.

At high thermal exposure (80–141 °C), WCRMO-2 diet was made using a flash sterilization system (Frontier Scientific Services). Frontier’s sterilizer consists of a circulating system of processing tubes that alternates between linear stretches of tubing and coiled spiraltherms that act as heat exchangers (Figure 1). To make 10 L of WCRMO-2 diet, 1.49 Kg of WCRMO-2 dry mix (WCRMO-2, Frontier Scientific Services) and 158 g of agar (7060, Frontier Scientific Services) were added to 9.26 L of cool tap water (~21 °C). The diet mixture was then pumped into the process lines from a tank and controlled via a metering pump that manages line pressure and flowrate of the product. The time of thermal exposure was set by manipulating the flowrate. The first heat exchanger was jacketed with hot oil set to a desired temperature. As the product flows through the coiled spiraltherm tubing, it rapidly gains thermal units from the hot oil jacketing the line. After departing the heated coil, the process line enters a series of linear switch-backs so that some excess heat is lost to atmosphere before reaching the cooling spiraltherm. At this point, the product enters a heat exchanger jacketed in cold (room temperature) water to rapidly lose thermal units and reach a desired dispense temperature. At the dispense temperature of 60 °C, 289.5 mL of 10% KOH (*w*/*v*) (F7633, Frontier Scientific Services) was added into the diet mixture to adjust the pH of the diet to 9, and the resulting diet solution was blended for 30 s to mix thoroughly. Subsequently, the diet solution was dispensed into a 96-well plate (3370, Corning Inc., Corning, NY, USA) using a repeater pipette (200 µL per well) in a biological cabinet (Forma 1800 Series Clean Bench, Thermo Scientific). The diet plate was then allowed to evaporate excess moisture for 10 min, stored in a refrigerator at 7 °C, and used for assays within 3 days.

At mild thermal exposure (50–80 °C), WCRMO-2 diet was made using WCRMO-2 dry mix (WCRMO-2, Frontier Scientific Services) according to the manufacturer’s procedure [27]. To prepare 1 L of WCRMO-2 diet, agar (15.8 g) was added to 926 mL of purified water, and the solution was brought to a full boil using a microwave (Hamilton Beach) until agar was completely melted. The agar solution was then transferred to a blender placed in a biological safety cabinet (SG403, SterilGARD^®^ III Advance cabinet, Sanford, ME, USA). When the agar solution cooled to designed temperatures (Table 1), 148.9 g of WCRMO-2 dry mix was added, and the mixture was blended for 10 s to mix thoroughly. Subsequently, 28.95 mL of 10% KOH (*w*/*v*) (P250, Fisher Scientific, Fair Lawn, NJ, USA) was added to increase the pH of the diet to 9, and the resulting diet solution was blended for 10 s to mix thoroughly. The diet was poured into a 1-L glass beaker containing a stir-bar and placed on a stirring hot plate (Cimarec^TM^, Thermo Scientific) set at the designed temperatures. The temperatures of diet solution were monitored using an infrared thermometer (IR002, Ryobi, Fuchu, Hiroshima, Japan) and held at the test temperatures for the designed times (5–30 min) using the hot plate. Subsequently, the diet solution was dispensed into a 96-well plate (3370, Corning Inc.) using a repeater pipette (200 µL per well), allowed to evaporate excess moisture for 10 min, stored in a refrigerator at 4 °C, and used for assays within 3 days.

### 3.1. Insect Artificial Diet Bioassays

The diet bioassays were conducted as described previously [25]. All materials used in the diet assays were surface-treated via exposure to UV light for 10 min in a biological cabinet (SterilGARD^®^ III Advance cabinet). Each diet treatment, which is WCRMO-2 diet made at different temperatures and time of thermal exposure (Table 1), was randomly assigned to a 12-well row of the 96-well plate and replicated at least 3 times in different diet plates. Each well was infested with one WCR neonate (<24 h old) using a fine paintbrush. A sealing film (TSS-RTQ-100, Excel Scientific, Inc., Victorville, CA, USA) was used to cover the plate. For ventilation, a hole was made in the sealing film over each well using a number zero insect pin. The plates were kept in an incubator (Percival, Perry, IA, USA) at 25 °C in darkness for 10 days. Larval molting was recorded daily during the experiments, whereas larval weight, survival, and evidence of contamination were recorded at the end of the experiments. For larval dry weight, all live larvae in each treatment were pooled per replicate (12 possible) into 95% ethanol, dried in an oven (Binder GmbH, Tuttlingen, Germany) at 55 °C for 48 h, and weighed using a micro balance (MSU6.6S-000-DM, Sartorius Lab Instruments GmbH & Co. KG, Goettingen, Germany).

### 3.2. Data Processing and Statistical Analyses

Survival and molting data were calculated by dividing the number of live larvae and successful larval molt from 1st to 2nd instar and from 1st to 3rd instar per replicate, respectively, by the initial number of larvae infested and multiplying by 100 to obtain percentages. Weight per larva (mg) was determined by dividing the dry weight by the number of larvae that survived per replicate.

For the experiment with high temperatures (80–141 °C) and short time of thermal exposure, the diet treatments that resulted in a “soft-set” of the media were excluded in the analyses because the soft-set form led to the death of the majority of larvae after 4 days post infestation. Because of the exclusion of these treatments, the remaining data points were not adequate to generate the response surface models of the measured responses. The remaining data were analyzed as a completely randomized experiment. The data were analyzed with analysis of variance (ANOVA) using PROC MIXED in SAS 9.4 (SAS Institute, Cary, NC, USA). Diet was the fixed effect, and replication was the random variable. Differences between the remaining treatments were determined using Fisher’s least significant difference (LSD) at *p* < 0.05. The percent variables (survival and molting) were arcsine square-root transformed prior to the analysis to meet assumptions of normality and homoscedasticity, whereas untransformed data were presented as mean ± SEM.

For the experiment with mild temperatures (50–80 °C) and extended time of thermal exposure, polynomial equations were generated to describe the impact of two factors (temperature and time of thermal exposure) on the measured responses (larval weight, molting, and survival). The best fit model for each measured response was selected from all possible models from linear to quartic polynomials generated with Design Expert^®^ (Stat-Ease, Inc., Minneapolis, MN, USA). Model selection was based on several criteria, including low model *p*-value, lack of fit *p*-value, low standard deviation, high R-values, and a low PRESS value [38,39]. Once more than one satisfactory model was generated, adequacy tests were performed to further evaluate the selected model, as described previously [40].

## 4. Results

### 4.1. Diet Quality with High Thermal Exposure and Short Time of Thermal Exposure

**Figure 2:**
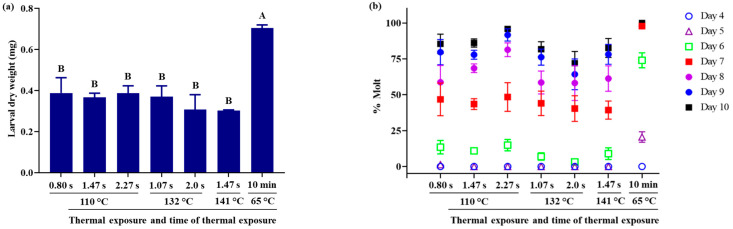

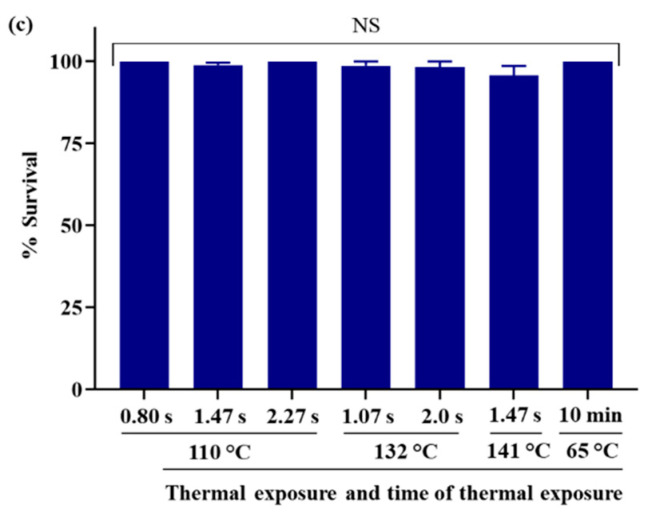
Effects of high thermal exposure (110–141 °C) and short time intervals of thermal exposure (0.8–2.3 s): dry weight (**a**), percent successful completion of molt to 2nd instar (**b**), and percent survival (**c**). Western corn rootworm larvae were reared on WCRMO-2 diet for 10 days. Means with bars followed by different letters are significantly different (*p* < 0.05). Means ± SEM.

Exposure to high temperatures (110–141 °C), even if constrained to a small window of time (0.9–2.3 s), had significant deleterious effects on the quality of WCRMO-2 diet for feeding WCR larvae. The WCRMO-2 diet exposed to the high temperatures for the brief time exposure resulted in larval weight significantly smaller compared to the control diet, WCRMO-2 diet made at a temperature of 65 °C for 10 min (*p* < 0.0001, *F_6,13_* = 15.88. Figure 2a). Average larval dry weight on the diet treatments ranged from 0.30 mg to 0.39 mg, while average dry weight on the control diet was 0.71 mg, an approximately 2-fold difference. There was no significant difference in dry weight on all diet treatments. Significant delays in larval molt to the 2nd instar were observed when they were reared on all diet treatments compared with the control diet (Figure 2b). There were significantly fewer 2nd instar larvae on the diet treatments than on the control diet after 5 days post infestation when larvae began to molt to 2nd instar. At day 5 post infestation, 20.6% of larvae had molted to 2nd instar on the control diet, whereas nearly 0% of 2nd instar larvae on all diet treatments (*p* < 0.0001, *F_6_,_32_* = 37.60). At day 7 post infestation, nearly 100% of the larvae had molted to 2nd instar on the control diet, significantly higher than that of all diet treatments (*p* < 0.0001, *F_6_,_32_* = 19.82). There was no significant difference in percent larvae molted to 2nd instar in the diet treatments (range from 39.3%–48.4%) at day 7 post infestation. No larvae that had molted to the 3rd instar were observed on the diet treatments by 10 days. Larval survivorship on all diet treatments ranged from 95.7% to 100%, which was not significantly different from survivorship on the control diet (*p* = 0.7764, *F_6,13_* = 0.53, Figure 2c).

### 4.2. Diet Quality with Mild Thermal Exposure and Extended Time of Thermal Exposure

**Figure 3:**
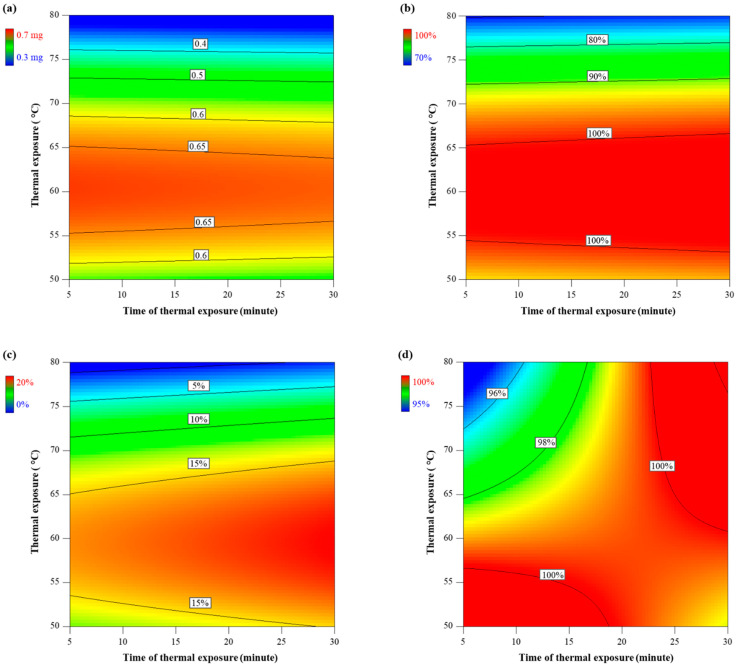
Effects of mild thermal exposure (50–80 °C) for extended times of thermal exposure (5–30 min). Contour plots of dry weight (**a**), percent successful completion of molt to 2nd instar (**b**) and percent successful completion of molt to 3rd instar (**c**), and percent survival (**d**). Western corn rootworm larvae were reared on WCRMO-2 diet for 10 days. Color bars display the magnitude of the measured responses.

The two-level factorial experiment yielded significant response surface models for larval weight (*p* < 0.0015, *F_3,7_* = 16.27), molt to 2nd instar (*p* = 0.0410, *F_3,7_* = 4.76), molt to 3rd instar (*p* = 0.0100, *F_3,7_* = 8.46) and a marginally significant model for survival (*p* = 0.0531, *F_3,7_* = 4.23) (Table 2). Models for weight, molt to 3rd instar, and survival had insignificant lack of fit, whereas there was a significant lack of fit for molt to 2nd instar due to a very small value of pure error. The relationships between the two factors tested (mild thermal exposure and extended time of exposure) and the quality of WCRMO-2 diet were revealed in contour plots (Figure 3). The contour plots generated from the models of responses measured (weight, survival, and molting) displayed the performance of larvae when reared on the WCRMO-2 diet prepared at the mild temperatures for the extended duration. The magnitude of the response variables is coded in color and can be envisioned as perpendicular to the page, as indicated by labelled isobars.

**Figure 4:**
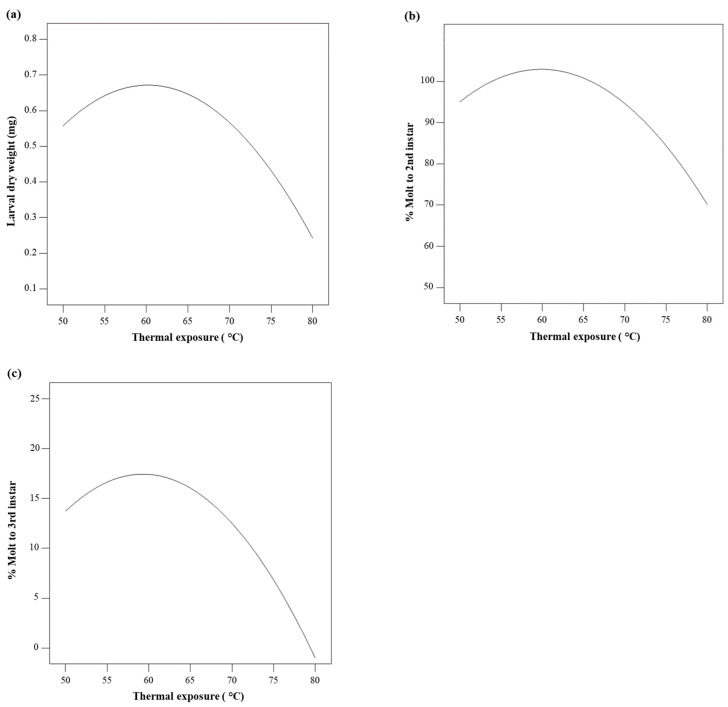
Nonlinear effects of mild thermal exposure (50–80 °C) for 15 min on dry weight (**a**), percent successful completion of molt to 2nd instar (**b**), and percent successful completion of molt to 3rd instar (**c**). Western corn rootworm larvae reared on WCRMO-2 diet for 10 days.

Models for weight, molt to 2nd instar, and molt to 3rd instar revealed that temperature had significant effects on these measured responses, while no significant effect on weight and molt was observed due to time of thermal exposure. *p* values of temperature were 0.0012, 0.0224, and 0.0051 in the models for weight, molt to 2nd instar, and molt to 3rd instar, respectively (Table 2). Non-linear effects of temperature on weight, molt to 2nd instar, and molt to 3rd instar were found (Figure 4). WCRMO-2 diet prepared a temperature of approximately 60 °C yielded the maximum larval weight and percent molt, whereas there were significantly negative effects on weight and molting when WCRMO-2 diet was produced at temperatures above 75 °C (Figure 3a–c and Figure 4).

A model for survival indicated marginally significant effects of time exposure (*p* = 0.0582) and the interaction between thermal exposure and time exposure (*p* = 0.0596). However, larval survivorship on all diet treatments was >95% (Figure 3d). A similar pattern was found in the experiment with high thermal exposure and short time of thermal exposure (Figure 2c). Consequently, survival was not considered as an important criterion for evaluation of the effects of the treatments.

### 4.3. Contamination

All experiments had minor contamination (<3%) except for diet treatments that experienced a soft-set of the media that were excluded from the analyses. No evidence for a relationship between contamination and two experimental factors (thermal exposure and time of thermal exposure) was determined. Similarly low contamination rates were observed previously [27].

## 5. Discussion

Published WCR diets require antibiotics as diet preservatives to alleviate bacterial contamination [24,25,27]. However, ingestion of antibiotics results in changes in WCR gut microbiota [31], thereby possibly interfering with the determination of the susceptibility of WCR to insecticide toxins using diet bioassays. The availability of a WCR diet free of antibiotics would facilitate research programs of this important pest. In diet processing, heating is required as one of the most important parts for activating gelling agents (e.g., agar) to stabilize diets and promote the form of suitable textures for insect feeding. This can be also used as an extremely effective means for destroying microbial contaminants derived from diet ingredients and preparation [34]. To further the goal of developing a diet free of antibiotics for WCR, we explored the effects of the thermal exposure from 50–141 °C for the short time (<3 s) and extended time (10–30 min) on the quality of WCRMO-2 diet based on life history parameters of WCR larvae fed on the treated diets. By using geometric and mathematical approaches, we further characterized the influence of both thermal exposure and time of thermal exposure, allowing determination of the optimum conditions (temperature and time) for making WCRMO-2 diet.

Our results indicated that the exposure to the high thermal exposure (110–141 °C) for brief intervals (0.9–2.3 s) caused detrimental effects on the performance of larvae on WCRMO-2 diet, indicating a possible reduction in nutrients required for WCR growth and development due to the high thermal exposure even if constrained for the short duration. WCR larvae fed on the treated diets exhibited significant reductions in weight and molting compared to those fed on the control diet made at the mild temperature of 65 °C for 10 min, whereas no difference in larval survivorship was observed between the diet treatments and the control diet. Heating is known to provide many benefits, including destruction of microbial contaminants, activation of gelling agents, increasing protein digestibility, denaturation of digestive inhibitors and harmful enzymes (e.g., phenol oxidases, lipo-oxygenase), increasing flavor, and acceleration of desirable chemical reactions [34]. However, severe overheating can cause reduction of protein digestibility, nutrient destruction (e.g., ascorbic acid, unsaturated lipid), destruction of vitamins, and formation of complexes (sugar amino acid products) [41]. A similar pattern of negative effects of heating on the quality of insect diets was previously reported for a diet of *Chrysoperla carnea* Stephens (Neuroptera: Chrysopidae) [42]. Initially, Vanderzant [43] developed a liquid diet for *C. canea* larvae that contains soy and casein hydrolysates as the main protein sources along with fructose, vitamins (ascorbic acid, B-vitamins), and other diet ingredients. This formulation was successfully used for the production of 18 generations of *C. canea*, but it did not promote the growth of *C. canea* after autoclaving at 121 °C [42]. The author successfully developed an improved diet that can be autoclaved by removing ascorbic acid (a heat-sensitive ingredient), replacing fructose with sucrose, and adding yeast hydrolysate and casein. These substitutions effectively compensated the loss of heat-sensitive nutrients. Only minor difference in percent adult recovery from larvae between the improved diet with and without autoclaving, which was 39% and 34%, respectively, was observed [42]. However, there was approximately 2-fold reduction in the percentage of adult recovery from larvae when *C. canea* larvae were reared on the standard diet [43] compared with the heat sterilized diet [42]. Griffin et al. [44] compared percent adult recovery from larvae of the boll weevils, *Anthonomus grandis* Boheman (Coleoptera: Curculionidae), reared on artificial diets that were made high temperatures of 130 °C, 138 °C, 144 °C, and 151 °C for 30 s using a flash-type sterilizer. They found that no significant effects of these temperatures on the percentage of adult recovery from larvae, though the highest temperature of 151 °C yielded fewer and smaller weevils than other temperatures. Our ongoing efforts to develop a heat-sterilized diet for WCR facilitating fewer or no antibiotics focus on the identification of alternative ingredients (e.g., yeast hydrolysate, casein) that can be compensated for the loss of nutrition in WCRMO-2 diet due to the overheating.

With diet ingredients in WCRMO-2 formulation, we demonstrated that a temperature of 60 °C yielded the highest larval performance on WCRMO-2 diet, while a time exposure of less than 30 min did not have significant impacts on the quality of WCRMO-2 diet. The exposure of WCRMO-2 diet to temperatures below 55 °C or above 65 °C resulted in a reduction in larval weight and molting compared to WCRMO-2 diet made at the optimum temperature. The significant losses in nutrients needed for WCR larval growth and development were observed when the WCRMO-2 diet was heated over 75 °C. Although most insect diets are often made at temperatures of 65–70 °C [24] that are likely used to avoid the loss of nutrition of heat-sensitive ingredients (e.g., ascorbic acid, vitamins, lipids), no information on the effects of relative low temperatures (50–80 °C) on insect diets is available. This study adds to the limited number of studies characterizing the effects of thermal exposure and time of thermal exposure, especially at mild temperatures, on insect diets. Some insect diets that have been reported to be heat-tolerant are usually produced at temperatures of 121 °C for 15–20 min by autoclaving or 141 °C for a few seconds by flash sterilizing [37,44]. It is noteworthy that WCR is nearly monophagous on maize roots and can survive on a few grass species [45]. This pest may require specific nutrients, and their bioavailability in WCRMO-2 diet is significantly reduced when the diet is exposed to temperatures over 75 °C.

Diet assays determining susceptibility of corn rootworm larvae typically utilizes a 96-well microtiter plate format [46,47,48]. Typically, each well is filled with 200 µL of diet and overlaid with toxins and followed by an infestation of a single neonate larva and sealing the well. This assay involves a labor-intensive filling process. A high-throughput system designed to run and analyze assays on a large scale, factorially increasing the number of compounds screened in the case of discovery, or the number of assays completed in the case of insect resistance management (IRM) studies would greatly facilitate research programs of this important pest. Research with lepidopteran insects has shown the great value of high-throughput systems for mass production and diet bioassays. In fact, utilizing a flash sterilizer coupled to a form-fill seal machine allowed the mass production of 3 million corn earworm pupae, *Helicoverpa zea* (Boddie) (Lepidoptera: Noctuidae), annually [49]. More recently, the high-throughput system can produce 25,000 rearing units per hour containing artificial diet and eggs of *Trichoplusia ni* (Hubner) (Lepidoptera: Noctuidae) through the use of automation [37]. For these systems, in addition to a flash sterilizer and a form-fill seal machine, a heat-sterilized diet is one of the key components. WCR has proven to be one of the most challenging pests in North America [50]. Many management strategies have been developed for controlling WCR (crop rotation, soil insecticides, Bt maize), but this pest has evaded nearly all management tactics in recent years [10,11,12,13,14,15,16,17]. Recently, WCR has been reported to evolve resistance to the newest management technology, RNA interference (RNAi) [51]. Future research could aim to leverage automation and robotics technology to establish the high-throughput system for WCR that would accelerate discovery efforts related to novel insecticide compounds and their related products.

## Figures and Tables

**Table 1:** Diet treatments used in 2-factorial experiments to rear western corn rootworm larvae.

Treatment	High Temperature		Mild Temperature	
	Temperature (°C)	Time Exposure (Second)	Temperature (°C)	Time Exposure (Minute)
1	141	1.47	80	17
2	132	1.07	76	26
3	132	2.00	76	10
4	110	0.80	65	30
5	110	1.47	65	17
6	110	1.47	65	17
7	110	1.47	65	17
8	110	2.27	65	5
9	88	1.07	55	26
10	88	2.00	55	9
11	80	1.47	50	17
12 (control)	65	600	65	10

**Table 2:** *p*-values, regression coefficients, and response surface-model fitting diagnostic statistics for western corn rootworm responses to a 2-factorial experiment at mild thermal exposure (50–80 °C) and extended time of thermal exposure (5–30 min). A: time of thermal exposure, B: temperature.

	Weight *p*-Values	Regression Coefficients	% Molt to 2nd Instar *p*-Values	Regression Coefficients	% Molt to 3rd Instar *p*-Values	Regression Coefficients	Survival *p*-Values	Regression Coefficients
Model	0.0015	-	0.0410	-	0.0100	-	0.0531	-
A	0.8538	−0.0005	0.8940	0.0005	0.5604	0.0098	0.0582	−0.0088
B	0.0012	0.1321	0.0224	0.0969	0.0051	0.0511	0.1563	−0.0033
AB	-	-	-	-	-	-	0.0596	0.0002
B^2^	0.0025	−0.0011	0.0478	−0.0008	0.0204	−0.0004	-	-
Lack of fit	0.1953		0.0005		0.9195		0.5496	
Model type	Quadratic (reduced)		Quadratic (reduced)		Quadratic (reduced)		Two-factor interaction	
R^2^	0.8745		0.6710		0.7838		0.6443	
R^2^_adj_	0.8208		0.5300		0.6911		0.4919	

## Data Availability

Not applicable.
